# PAX6, modified by SUMOylation, plays a protective role in corneal endothelial injury

**DOI:** 10.1038/s41419-020-02848-5

**Published:** 2020-08-12

**Authors:** Fei Yu, Weijie Zhang, Chenxi Yan, Dan Yan, Meng Zhou, Junzhao Chen, Xiangteng Zhao, Aoxue Zhu, Jie Zhou, Huiqing Liu, Hao Sun, Yao Fu

**Affiliations:** 1 Department of Ophthalmology, Ninth People’s Hospital, Shanghai Jiao Tong University School of Medicine, Shanghai, 200011 China; 2 Shanghai Key Laboratory of Orbital Diseases and Ocular Oncology, Shanghai, 200011 China; 3 Department of Biochemistry and Molecular Cell Biology, Shanghai Key Laboratory for Tumor Microenvironment and Inflammation, Shanghai Jiao Tong University School of Medicine, Shanghai, 200025 China; 4 Department of Pediatric Neurosurgery, Shanghai Children’s Medical Center, Shanghai Jiao Tong University School of Medicine, Shanghai, 200127 China

**Keywords:** Mechanisms of disease, Sumoylation, Sumoylation, Trauma

## Abstract

Treating corneal endothelial diseases tends to be challenging as human corneal endothelial cells (CECs) do not proliferate in vivo. The pathogenesis or mechanisms underlying injured CECs need further studies. The abnormal expression of PAX6, which is an essential transcription factor for corneal homeostasis, exhibits corneal endothelial defects. However, the effects of PAX6 protein involved in corneal endothelial wound process are still unknown. Here, we found the upregulated protein levels of PAX6 in human corneal endothelial monolayer after injury; the expression of PAX6 also increased in murine and rat corneal endothelium injury models. Enforced PAX6 expression could alleviate the damages to CECs via regulating permeability by prompting cellular tight junction. In addition, SUMOylation mainly happened on both K53 and K89 residues of 48-kD PAX6 (the longest and main isoform expressed in cornea), and de-SUMOylation promoted the stability of PAX6 protein in vitro. In CECs of SENP1^+/−^ mice, increased SUMOylation levels leading to instability and low expression of PAX6, delayed the repair of CECs after injury. Furthermore, overexpression of PAX6 accelerated the rate of corneal endothelial repair of SENP1^+/−^ mice. Our findings indicate that SENP1-mediated de-SUMOylation improving the stability of PAX6, amplifies the protective effects of PAX6 on corneal endothelial injuries, highlighting potentials of PAX6 and/or SUMOylation to be used as a treatment target for corneal endothelial disorders.

## Introduction

Corneal endothelial cells (CECs) locate at the innermost layer of the cornea, which are essential for corneal transparency due to its barrier and ionic “pump” function^1^. The cell cycle of human CECs (hCECs) is arrested at the G1 phase, and hCECs cannot divide in vivo^2,3^. Under normal conditions, though there is an average loss rate of 0.3–0.6% CECs per year, adjacent cells may cover the wound area by spreading and/or migrating. Therefore, CECs can maintain the balance between fluid inlet and outlet, preserving corneal transparency in a compensatory manner^4,5^. Yet, extra traumas, corneal surgeries, and stresses from glaucoma or endothelial dystrophies may significantly reduce endothelial cell density, even leading to decompensation of residual CECs^6^. When hCEC density drops to less than 500–1000 cells/mm^2^, surplus storage of aqueous humor in the corneal stroma may cause corneal edema, bullous keratopathy, and even loss of visual acuity^3,7^. Currently, corneal transplantation is the only definitive treatment for patients with corneal endothelial decompensation. However, the worldwide shortages of donor corneas, highly technology dependence of surgical procedures, and numerous postoperative complications, such as immunologic rejection, highlight the urgency of alternative treatments for corneal endothelial injuries and the importance of insights into corneal endothelial diseases^8,9^. Previous studies have shown that topical Rho kinase (ROCK) inhibitors can promote the healing process of cryoinjured corneal endothelium in animal models^10,11^. Y-27632, a ROCK inhibitor, has been experimentally applied in bullous keratopathy patients during cultured hCEC transplantation^12^. However, to date, due to the risk of endothelial–mesenchymal transition (EMT) and the obstacles in ex vivo expansion of human CECs, no pharmaceutical and novel grafting therapies, such as tissue engineering corneas, can solve the needs of patients with corneal endothelial decompensation^3,13,14^. Therefore, to explore new ideas for therapeutic approaches, the intrinsic mechanisms and the critical factors in the process of corneal endothelial injury need to be elucidated.

PAX6, an evolutionally conserved transcription factor, is critical for healthy development and homeostasis of the anterior segment, especially the cornea^15,16^. PAX6 protein has four isoforms, named p48, p46, p43, and p32, respectively, with the referring molecular weights of 48, 46, 43, and 32 kD^17^. In the cornea, p48 and p46 PAX6 were the main isoforms to exist^18,19^. After birth, PAX6 protein is restricted in human corneal and limbal epithelium, and inadequate levels of PAX6 protein can induce abnormal differentiation of corneal limbus and delayed healing of injured corneal epithelium^20,21^. PAX6^+/−^ adult mice exhibited severe corneal endothelial defects, while the potential effects of PAX6 protein on postnatal CECs are still unknown, and the PAX6-involving mechanisms underlying corneal endothelial diseases need to be further studied^22^.

SUMOylation, as one of the important post-translational modifications, has emerged widely and been implicated in numerous key cellular functions, including cell cycle, DNA repair, transcription, and epigenetic regulation, through regulating subcellular localization, stability, or activity of the target proteins^23–26^. SUMO (a small ubiquitin-like modifier) is a 11-kD protein that can be covalently attached to lysine residues in substrate proteins via an enzymatic cascade^27,28^. In the eye, SUMOylation and sentrin/SUMO-specific protease 1 (SENP1) protein are detected in many cell lines from the murine anterior segment, and SUMOylation modification has been reported to involve in corneal epithelial homeostasis^24,29,30^. SUMOylation of p32 PAX6 has been confirmed in vitro via protein synthesis system^18,29^. However, SUMOylation modification of p48 PAX6, the longest and main isoform in the cornea, has not yet been confirmed in the cellular expression system, and the effects of SUMOylated PAX6 in ocular tissue, especially corneal endothelium, are worth exploring further.

## Materials and methods

### Human corneal endothelial monolayer isolation

Human corneolimbal rims stored at 4 °C in Optisol (Chiron Vision, Irvine, CA) were obtained from donor corneas, which were used for corneal transplantation. The corneal endothelium monolayer was torn off from the rim for further staining analysis. The Ethics Committee of Shanghai Ninth People’s Hospital approved the study.

### Scratch assay

Authenticated human immortalized CEC line, B4G12 cells were purchased from the Creative Bioarray Company (Shirley, NY, USA) and cultured in Dulbecco’s modified Eagle’s medium (DMEM) (HyClone, Utah, USA) supplemented with 10% fetal bovine serum. Wounds were created by scratching with a sterile pipette tip. B4G12 cells were collected at 6 and 12 h after scratches for western blot analysis.

### Construction of corneal endothelium injury model

All 8-week-old male BALB/c mice and 6-week-old male Sprague Dawley rats used in the study were purchased from the Animal Laboratory, Shanghai Ninth People’s Hospital, Shanghai Jiaotong University School of Medicine, Shanghai, China. SENP1^+/−^ and WT mice were kindly provided by Yong Li (Shanghai Jiao Tong University School of Medicine, Shanghai, China). The Animal Care and Experiment Committee of the Shanghai Ninth People’s Hospital approved all experimental protocols. Furthermore, all procedures were performed according to the Association for Research in Vision and Ophthalmology (ARVO) Statement for the Use of Animals in Ophthalmic and Vision Research. Mice and rats were separately kept in colony room with a 12-h light/dark cycle at 25 °C for 7 days before initiating experiments. The sample sizes were in the range of the published literature, and exclusion criteria were general health status during the experiments. All animals were randomly divided into the control and injured groups. Corneal endothelial freezing injury model was established as previously described^31^. The investigators were blinded to group allocation during data collection and analysis.

### Clinical examinations

Corneal opacity was recorded with photographs using a slit-lamp microscope. The central corneal thickness (CCT), indicating the edema degree of the cornea, was evaluated by the anterior segment optical coherence tomography (OCT) (Carl Zeiss Meditec). CCT was measured thrice in each eye, and the average of the three readings was taken.

### Enforced expression of GFP-PAX6 via intracameral injection

We overexpressed PAX6 protein in the corneal endothelium of rats via adenovirus (Gemoneditech, Shanghai, China). Adenoviral particles express GFP-PAX6 (Ad-PAX6) or express the empty vector with GFP (Ad-GFP). The left eyes of rats were treated with Ad-GFP (5 μL, 1 × 10^10^ pfu, Control group) and the right eyes of rats were treated with Ad-PAX6 (5 μL, 1 × 10^10^ pfu, PAX6 group) via the intracameral injection with a 10-μL microsyringe (Hamilton, 1702RN, Reno, NV, USA) as previously described^32^. After 3 days, the corneal endothelial injury model was constructed following the above-reported protocols. After 24 h of cryoinjury, rats were sacrificed for staining analysis of adenoviral transfection efficiency, following clinical examinations of corneas.

### Rat corneal endothelial monolayer isolation

Rat corneas were isolated from normal or injured eyes in cold phosphate buffer saline (HyClone, Utah, USA). With the endothelium facing upward, the endothelial monolayers were torn off from the corneal tissues for protein extraction.

### Organ culture

For higher transfection efficiency, normal and injured mice corneas were isolated to be organ-cultured as previously described^33^. Ad-GFP (0.5 μL, 1 × 10^10^ pfu, Control group) or Ad-PAX6 (0.5 μL, 1 × 10^10^ pfu, PAX6 group) was added to the cultured injured corneas. After 24 h, tissues were collected for flat-mount corneal staining to evaluate CECs after overexpression of GFP or PAX6.

### Immunofluorescence staining

For staining of cross sections, eyes of mice and rats were embedded in Tissue-Tek Optimal Cutting Temperature compound (Sakura Seiki, Tokyo, Japan) and then cut into 8-μm sections along the direction of the optical nerve. Sections and HCE monolayer on the slides were fixed with 4% formaldehyde for 20 min and then blocked with 2% donkey serum albumin and 0.1% triton. Next, sections and monolayers were incubated with PAX6 antibody (9013101, Biolegend, San Diego, CA, USA) or SUMO1 antibody (4940, Cell Signaling Technology, USA) overnight at 4 °C, followed by CyTM3-conjugated secondary antibody (Jackson ImmunoResearch, PA, USA) for 60 min at room temperature. Counterstaining was performed with Hoechst 33342 (Invitrogen, California, USA). To compare the expression of PAX6 or SUMO1, Image-Pro Plus software (Media Cybernetics) was used to count fluorescence intensity per area of images taken by confocal (Zeiss Confocal LSM 710 microscope) or fluorescence microscope (Olympus BX51). Three random fields were selected for each sample (*n* = 3/group).

For evaluating the patterns of the corneal endothelium, staining of flat-mounted corneas was used. Eyes of rats were first fixed with 4% formaldehyde overnight. Then corneas were isolated and blocked with 5% donkey serum albumin and 0.1% triton for 1 h. Next, corneas were incubated with ZO-1 antibody (ab221547, Abcam, MA, USA) overnight at 4 °C, also followed by CyTM3-conjugated secondary antibody and counterstaining as above. Last, corneas were mounted under a coverslip, and the endothelium was viewed and photographed. Three random fields were selected for each sample (*n* = 3/group).

### Plasmid transfection

Culturing and transfection of cells were performed as previously described^34^. In most of our experiments, equimolar ratios of DNAs were employed for cotransfection experiments, and the total amount of plasmid DNA was trimmed with an empty vector. A 1.2–1.5-fold amount of PAX6 WT plasmids was used for transfection in experiments of verifying SUMOylated residues and CHX assays to ensure that the wide-type and mutant transfectants expressed comparable levels of protein.

HA-PAX6 and Flag-PAX6 were purchased from Genomeditech (Shanghai, China), and Myc-SUMO2/3, Flag-SUMO1, RGS-SENP1, and RGS-SENP1m plasmids were previously described^34^. Flag-K53/89R PAX6, various Flag-PAX6 mutations (Flag-K28R, K53R, K69R, K89R, K100/K105R, K148R, K159R, K221/223R, K241R, K260R, K278R, or K284R PAX6), and HA-PAX6 mutations (HA-K28(1), K53(1), K69(1), K89(1), K100(1), K105(1), K148(1), K159(1), K221/223(2), K241(1), K260(1), K278(1), or K284(1) PAX6) were generated by a QuikChange Site-Directed Mutagenesis Kit (Stratagene, La Jolla, CA). Protein sequence data have been deposited in the NCBI protein database under accession number NP_001595.

### Immunoprecipitation

To exclude the numerous modifications, denaturing immunoprecipitations were performed as previously described^35^. These experiments were repeated at least three times for each condition.

### Cycloheximide (CHX)-chase assays

Protein synthesis was blocked by CHX (Sigma, St. Louis, MO, USA) as previously described^35^. In this experiment, a 1.2–1.5-fold amount of flag-tagged PAX6 (WT) plasmid compared with flag-tagged PAX6 (K53/89R) was also used for transfection. PAX6 expression was compared with Flag/GAPDH at 0 h.

### Western blot assay

PAX6 antibody (9013101, Biolegend, San Diego, CA, USA) was generated against only p46 and p48 PAX6. GAPDH antibody was from Abcam (ab8226, Abcam, MA, USA). Procedures and other antibodies were as previously described^34^. Bound antibodies were detected by DyLightTM680-conjugated secondary antibodies (Sigma, St. Louis, MO, USA), and the protein expression levels were observed using an Odyssey V 3.0 image scanner (LI-COR). These blots were repeated at least three times for each condition.

### Statistical analysis

Data are presented as mean ± standard error of the mean (SEM), and all experiments were repeated at least 3 times. The variance was homogeneous for groups that are being statistically compared. *p* Values were calculated by Student’s *t* test and two-way ANOVA followed by Bonferroni post test in GraphPad Prism 7.0 (GraphPad, San Diego, CA). A *p* value of <0.05 indicated statistical significance.

## Results

### The expression of PAX6 protein increased in injured human corneal endothelium monolayer and B4G12 cells

**Fig. 1:**
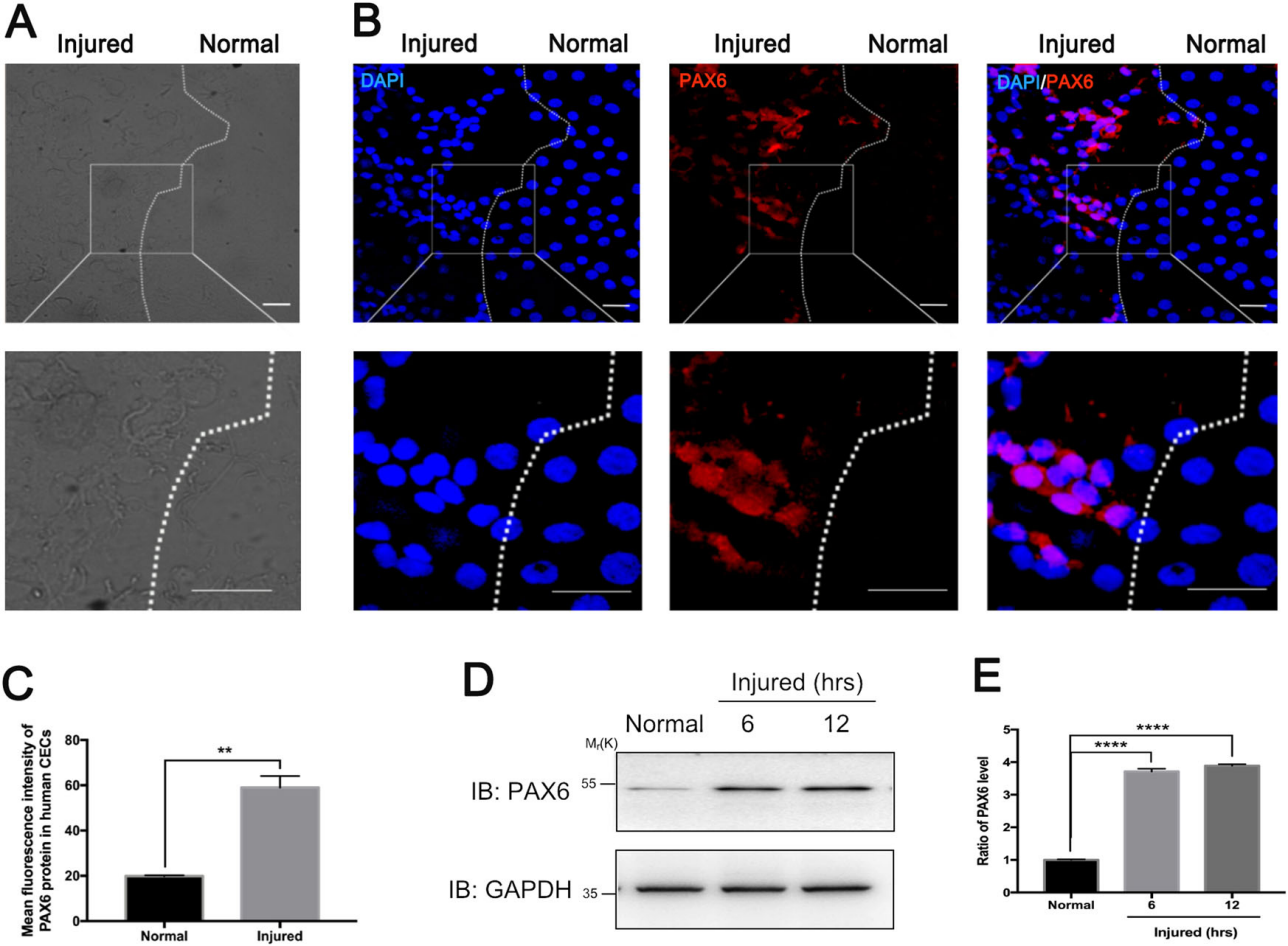
Injuries upregulate the expression of PAX6 protein in human corneal endothelial cells. **a**, **b** Human corneal endothelium (HCE) monolayer derived from the corneal ring after transplantation was employed for immunofluorescence staining. **a** Light microscopy images showed that the HCE monolayer could be roughly divided into the injured group (left), which appeared more irregular and rugged than the relative normal group (right). **b** Fluorescence micrograph depicted PAX6 protein expression in the injured and relatively normal hCECs. The dotted line highlighted the boundary between relative normal and wounded hCECs. The framed areas were magnified in the images below. **c** The averages of mean fluorescence intensity of PAX6 protein in relative normal and injured human CECs were compared. In total, 40–60 cells per group of three random fields were selected for statistics. **d**, **e** Human corneal endothelial cell line (B4G12) was cultured after scratches; western blot was used to analyze the PAX6 protein expression after injury. **d** Representative bands showed the expression level of PAX6 protein in normal and injured B4G12 cells at 6 and 12 h after injuries. GAPDH served as the loading control. **e** Densitometric analysis of specific immunoreactive bands. Scale bar = 20 μm. Error bars showed means ± SEM (*n* = 3; **p < 0.01, ****p < 0.0001).

To study the wounded human corneal endothelium (HCE), we utilized the HCE monolayer from the corneal ring, which was left after corneal grafting. As reported, the corneal ring could be seen as partially wounded due to the removal of the central cornea by trephination^36^. We observed the HCE monolayer through a light microscope and defined irregular and rough cells, which were close to the incision of trephinations, as the injured ones (left, injured part), and cells that appeared regular and intact, which were close to the limbus, as the relative normal CECs (right, normal part) (Fig. 1a).

To further examine the expression of PAX6 in the HCE monolayer, we employed immunofluorescence chemistry using PAX6 antibody that was generated against only p46 and p48 PAX6. The images of PAX6 staining showed that there was little PAX6 protein in relative normal CECs (right, normal part), which was also identified by relatively regular nuclei, while the expression of PAX6 protein remarkably increased in injured hCECs with irregular nuclei (left, injured part) (*p* < 0.01) (Fig. 1b, c).

To confirm whether injuries could upregulate PAX6 expression in CECs, B4G12 cells with scratch injuries were analyzed. Western blot assay and statistics showed that the expression levels of PAX6 were significantly increased at 6 and 12 h after scratching the B4G12 monolayer (*p* < 0.0001) (Fig. 1d, e). Taken together, PAX6 protein could hardly be detected in normal hCECs, while wounds could promote the expression of PAX6 protein in both primary HCE and HCE cell lines.

### PAX6 protein was upregulated in murine and rat corneal endothelial injury models in vivo

**Fig. 2:**
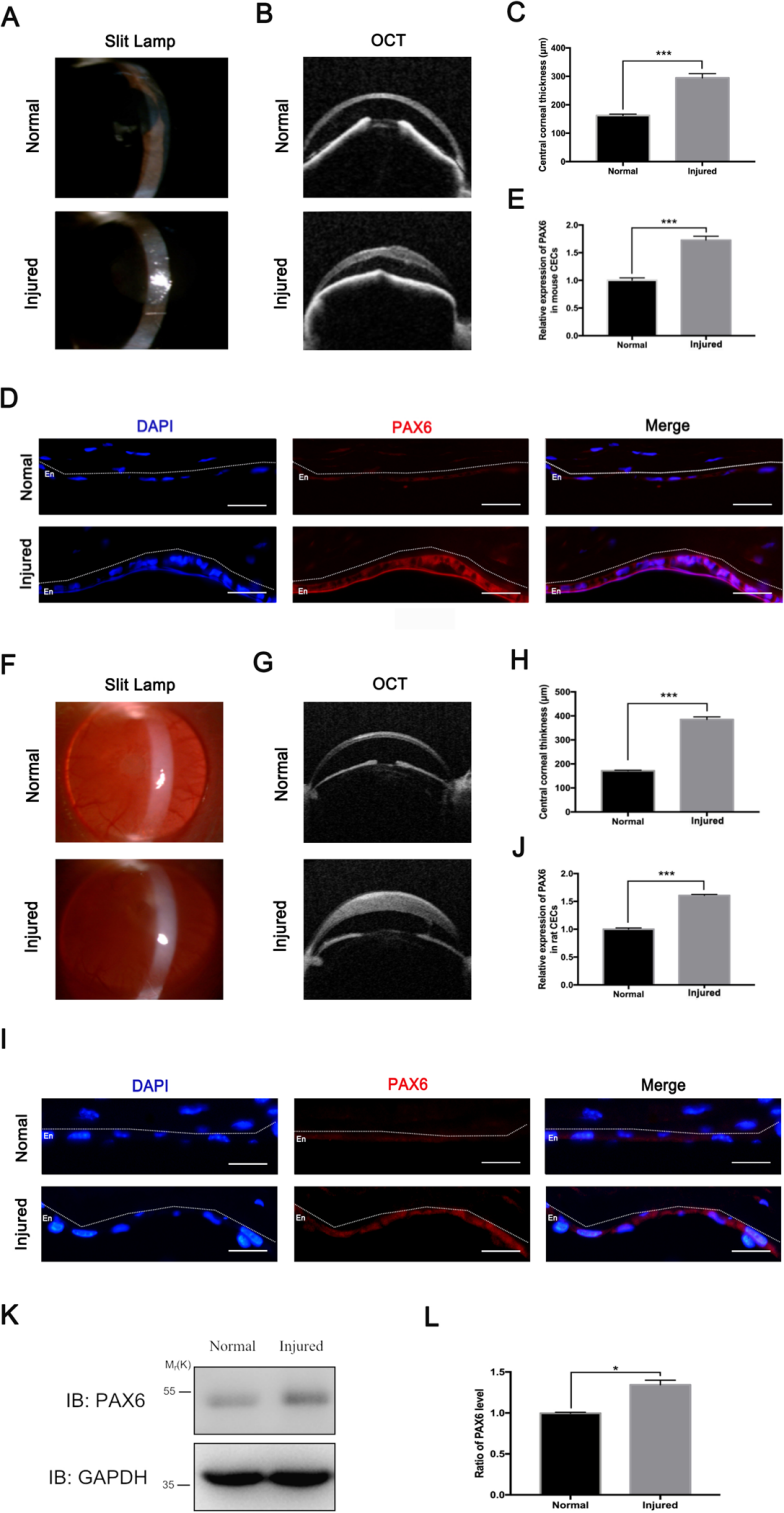
PAX6 protein in murine and rat corneal endothelium was activated by injuries in vivo. **a**–**e** A murine model of corneal endothelial wound was established by transcorneal cryogenic injury. **a**, **b** Representative images of slit-lamp microscope (**a**) and optical coherence tomography (OCT) (**b**) showed normal and edema corneas 24 h after cryoinjury. **c** Data on central corneal thickness were compared. **d** PAX6 staining of cross-sectional normal and injured corneas from mice were taken by a fluorescence microscope under the same parameters. **e** The relative expression of PAX6 protein in mice CECs was calculated. **f**–**j** A rat model of corneal endothelial wound was also established by transcorneal cryogenic injury. **f**–**g** Representative images of slit-lamp microscope (**f**) and optical coherence tomography (OCT) (**g**) showed normal and edema corneas 24 h after cryoinjury. **h** Data on central corneal thickness were compared. **i** PAX6 staining of cross-sectional normal and injured corneas from rats were taken by a fluorescence microscope under the same parameters. **j** Relative expression of PAX6 protein in rat CECs was evaluated. The dotted lines highlighted the boundary between the endothelium (En) and the anterior segment. **k** Representative WB showed that PAX6 expression increased in corneal endothelial tissues after injury. GAPDH served as the loading control. **l** The ratio of PAX6/GAPDH from injured CECs relative to that of normal CECs. Scale bar = 20 μm. Error bars showed means ± SEM (*n* = 3/group; *p < 0.05, ***p < 0.001).

A murine model of the wounded corneal endothelium was employed to prove the upregulation of PAX6 expression in CECs after injury in vivo. Slit-lamp microscope and OCT examinations indicated the apparent edema of corneas 24 h after the transcorneal cryogenic injury (Fig. 2a, b). Statistics showed that the average CCT of injured corneas was 1.8-fold compared with that of the normal corneas (Fig. 2c). After 24 h of cryoinjury, mice were sacrificed so as to collect the corneas for PAX6 staining. The representative fluorescent images showed that cryoinjuries induced the endothelial layer edema. Moreover, PAX6 protein was proved to apparently increase in injured mouse corneal endothelium in vivo compared with the normal mouse corneal endothelium (*p* < 0.001) (Fig. 2d, e).

We further confirmed the increased expression of PAX6 protein in injured CECs of rats in vivo. Consistent with the results of the murine model, slit-lamp microscope and OCT examinations showed the apparent corneal edema 24 h after cryoinjury (Fig. 2f, g). The average CCT of injured rat central corneas was approximately 2.25 times that of the normal cornea (Fig. 2h). PAX6 expression was significantly increased in injured rat CECs (*p* < 0.001) (Fig. 2i, j). In addition, western blot assay also indicated that PAX6 protein significantly increased in rat CECs after injury (*p* < 0.05) (Fig. 2k, l). To sum up, injuries induced the upregulation of PAX6 protein in human, mouse, and rat CECs, thus indicating that PAX6 protein conservatively participated in the injury process of CECs among different species.

### Enforced PAX6 alleviated corneal edema in wounded corneal endothelium in vivo

**Fig. 3:**
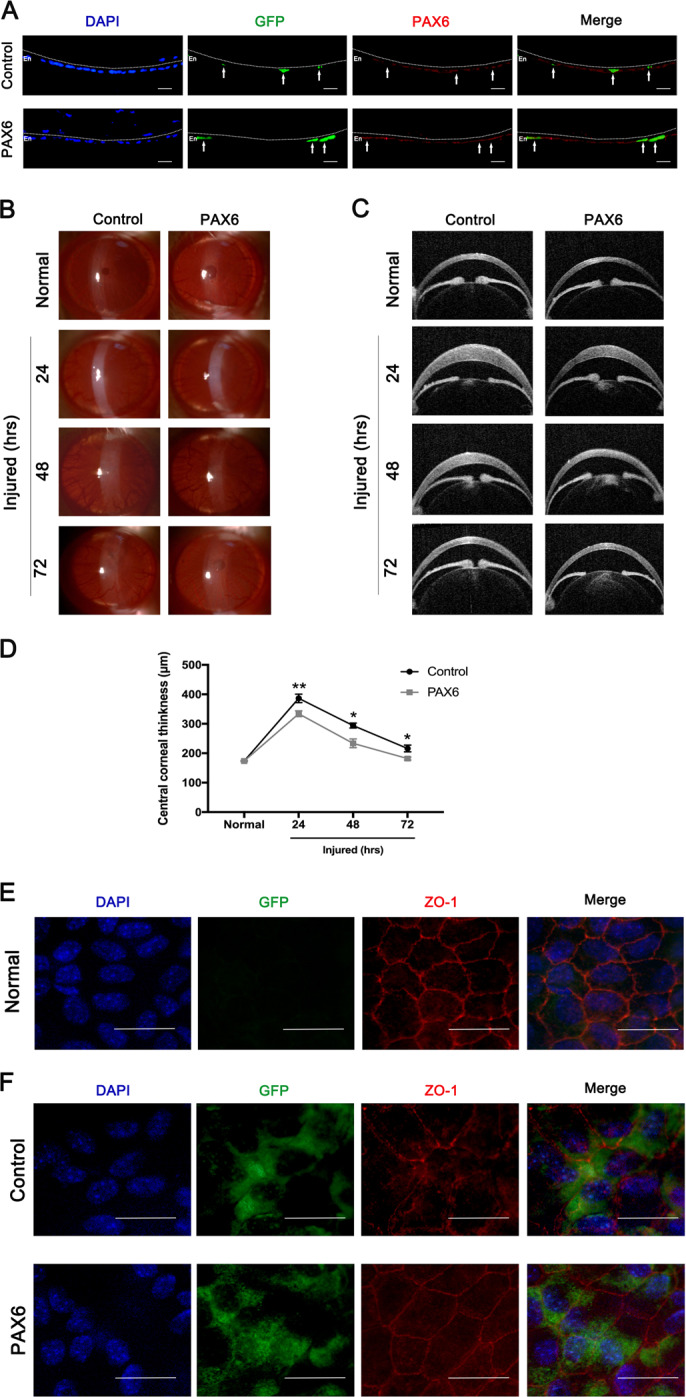
Enforced PAX6 protein in corneal endothelium alleviated corneal edema after injury in vivo. Adenovirus-expressing GFP-PAX6 (PAX6 group) or GFP vector (Control group) was separately injected into the anterior chamber of rat eyes. **a** GFP expression of corneal endothelium indicated the transfection efficiency of GFP-encoding vector and PAX6 adenovirus. White arrows showed that representative GFP^+^ cells had increased the level of PAX6 protein in the PAX6 group, but not the Control group. The dotted lines highlighted the boundary between the endothelium (En) and the anterior segment. **b**–**d** PAX6 protein was overexpressed in corneal endothelium of rats via adenovirus injection through the anterior chamber. Then, the rat corneal endothelial injury model was induced by cryoinjury 3 days after injection. **b**, **c** Representative images of slit-lamp microscope (**b**) and optical coherence tomography (OCT) (**c**) showed opacity and edema of corneas at 24, 48, and 72 h after injury. Note: Corneal opacity and corneal edema were lower in the PAX6 group, compared with the Control group. **d** Analysis of central corneal thickness in control and PAX6 groups at all investigated time points. **e**, **f** Normal and injured corneas were acquired for organ culture. GFP-vector and GFP-PAX6 adenovirus-transfected CECs in vitro and corneas were collected for ZO-1 staining. **e** Representative flat-mounted images of ZO-1 expression in the normal CECs. **f** ZO-1 staining of injured CECs from the Control and PAX6 groups. Note that PAX6 overexpression promoted tight junction among CECs after injury. Scale bar = 20μm. Error bars showed means ± SEM (*n* = 3/group; *p < 0.05, **p < 0.01).

To explore the effects of increased PAX6 protein in corneal endothelium after injury, we enforced PAX6 expression in rat corneal endothelium via intracameral injection of adenovirus GFP-PAX6 (PAX6 group), while adenovirus GFP vector was used as the control (Control group). The GFP protein expression of corneal endothelium confirmed the successful transfection: GFP-positive cells (white arrows) had increased expression of PAX6 than GFP-negative cells in the PAX6 group, while GFP-positive cells had the same expression of PAX6 as GFP-negative cells in the Control group (Fig. 3a). At 24, 48, and 72 h after cryoinjury, images of slit-lamp microscope (Fig. 3b) and OCT (Fig. 3c) indicated that the increased PAX6 expression in corneal endothelium alleviated the corneal opacity and edema. The averages of CCT of the PAX6 group were significantly lower than those of the control group at all investigated time points (Fig. 3d). Furthermore, ZO-1 staining of corneal endothelium showed that normal CECs had regular polygonal shapes (Fig. 3e), and at 24 h after injury, residual live CECs expanded to compensate (Fig. 3f). In addition, enforced PAX6 promoted the tight junction among cells compared with the Control group (Fig. 3f). Taken together, the results showed that PAX6 protein in corneal endothelium could be upregulated by injuries, while enforced PAX6 could reduce the corneal edema caused by the damage to the corneal endothelium via improving the “barrier” function. Therefore, PAX6 protein is essential in protecting corneal endothelium in the injured process of CECs.

### p48 PAX6 was modified by small ubiquitin-like modifier 1 (SUMO1), but not SUMO2/3

**Fig. 4:**
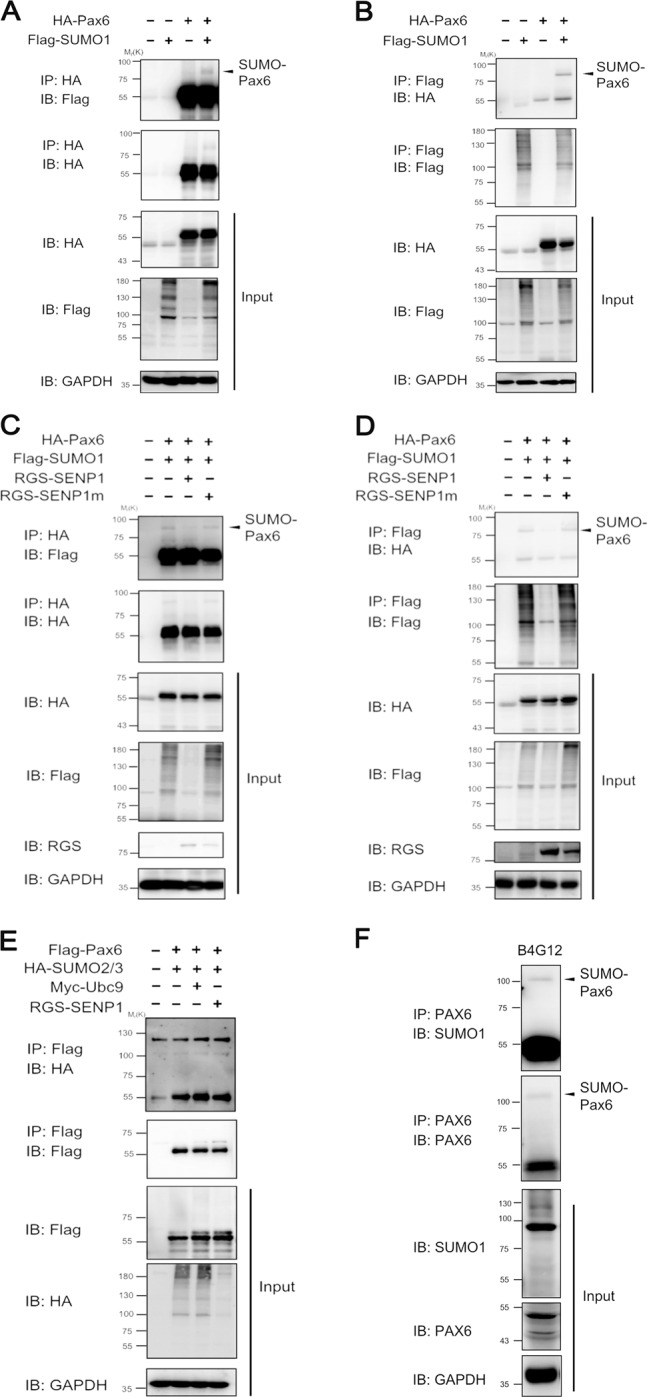
PAX6 is modified by SUMO1, but not SUMO2/3. **a**, **b** 293T cells were co-transfected with HA-tagged PAX6 and Flag-tagged SUMO1, as indicated. Whole-cell lysates were prepared 24 h post transfection under denaturing conditions and immunoprecipitated with anti-HA antibody (**a**) or anti-Flag antibody (**b**). Western blot analysis was used to detect SUMOylation-modified PAX6. **c**, **d** 293T cells were co-transfected with HA-tagged PAX6, Flag-tagged SUMO1, RGS-tagged SENP1, or RGS-tagged SENP1m as indicated. Whole-cell lysates were prepared 24 h post transfection under denaturing conditions and immunoprecipitated with anti-HA antibody (**c**) or anti-Flag antibody (**d**). Western blot analysis was used to detect de-SUMOylation of PAX6. **e** 293T cells were co-transfected with Flag-tagged PAX6, HA-tagged SUMO2/3, Myc-tagged Ubc9, or RGS-tagged SENP1 as indicated. Whole-cell lysates were prepared 24 h post transfection under denaturing conditions and immunoprecipitated with anti-Flag antibody. **f** Cell lysates of B4G12 were prepared under denaturing conditions and subjected to immunoprecipitation with anti-PAX6 antibody, followed by western blot using anti-PAX6, anti-SUMO1, or anti-GAPDH antibodies. The result of IP–WB showed the endogenous SUMOylation of PAX6.

Next, we explored how PAX6 protein exerted its protective role in the wounded CECs. SUMO modification is a vital process in the regulation of transcription factors. Previous studies have reported that in vitro system-synthesized p32 PAX6 protein can be modified by SUMO1, while in the cell system, the SUMOylation of p48 PAX6 protein, as the longest and main form of PAX6 in the cornea, has not yet been confirmed^18^. To systemically test SUMOylation modification of p48 PAX6, we transiently transfected 293T cells with hemagglutinin (HA)-tagged PAX6 plasmid, either alone or together with Flag-tagged SUMO1. Through the immunoprecipitation by HA, we found that the presence of a high-molecular-weight band on western blot corresponded to SUMOylated PAX6 only in the cells co-transfected with HA-PAX6 and Flag-SUMO1 (line 4) (Fig. 4a). Next, we demonstrated that the SUMO1-modified PAX6 band could also be detected on western blot by immunoprecipitating flag (line 4) (Fig. 4b). To further determine whether SUMO1-modified PAX6 could be regulated by sentrin/SUMO-specific protease 1 (SENP1), based on the previous transfection with HA-tagged PAX6 and Flag-tagged SUMO1, we additionally overexpressed wild-type SENP1 to completely abolish SUMOylated PAX6 (line 3), as well as a catalytically inactive mutant SENP1 (SENP1m) failed to deconjugate SUMOylated PAX6 (line 4) in either HA (Fig. 4c) or Flag (Fig. 4d) immunoprecipitation assays. Besides SUMO1, SUMO2/3 SUMOylation was also examined. 293T cells were transiently transfected with Flag-PAX6 with HA-tagged SUMO2/3, and the SUMOylation-conjugating enzyme UBC9 or SENP1 was extra overexpressed to further confirm SUMOylation (Fig. 4e). However, there was no high-molecular-weight band on western blot, indicating that PAX6 could not be SUMOylated by SUMO2/3. Furthermore, we also confirmed the endogenous SUMO1 modification of PAX6 protein in human CEC line, B4G12 by immunoprecipitating PAX6 (Fig. 4f). To sum up, these results indicated that p48 PAX6 was SUMOylated by SUMO1, while SENP1 acted as a major de-SUMOylating enzyme for p48 PAX6.

### SUMOylation of PAX6 predominately occurred at lysine 53 and 89 residues

**Fig. 5:**
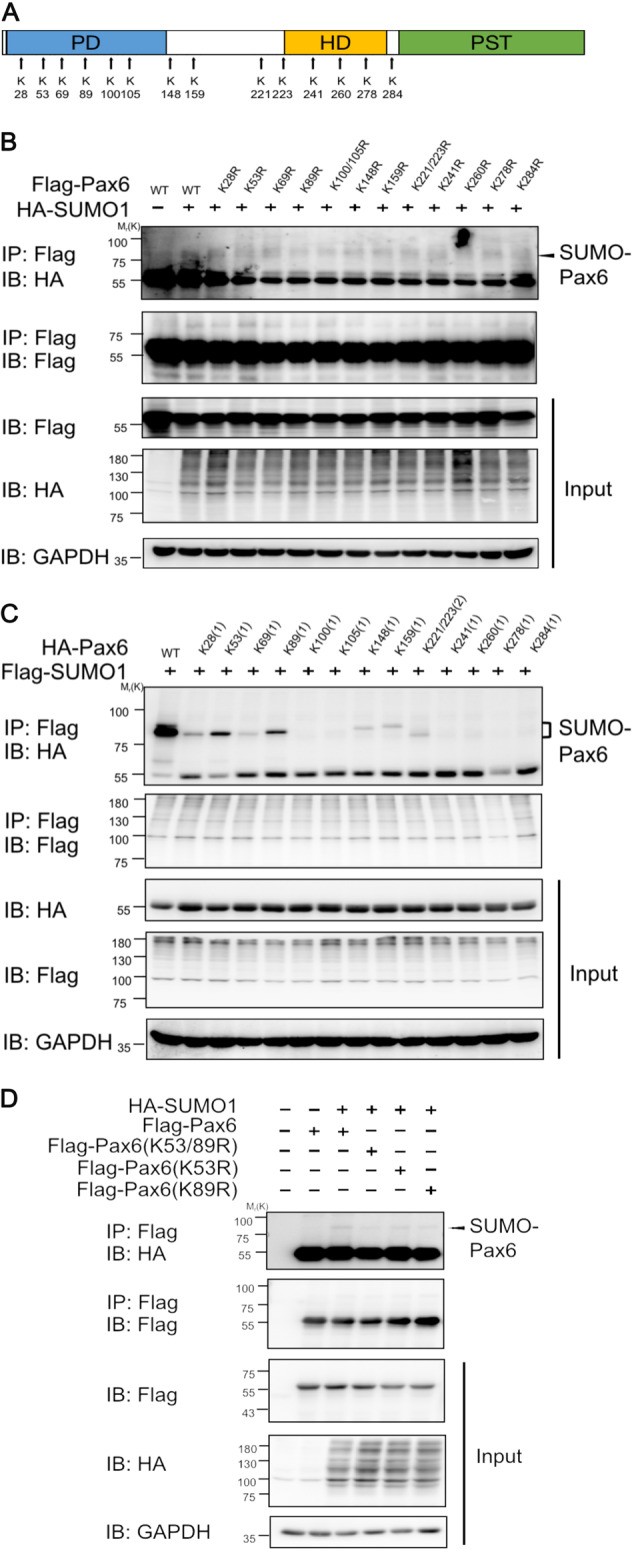
SUMO1 modification mainly occurred at lysine 53 and 89 residues of 48-kD PAX6. **a** Pattern diagram indicating the distribution of all lysine residues of 48-kD PAX6. **b** 293T cells were co-transfected with HA-tagged SUMO1 with the indicated wild-type PAX6 and PAX6 variants, containing one or two adjacent lysine (K) residues mutated to arginine (R) residues (K28R, K53R, K69R, K89R, K100/K105R, K148R, K159R, K221/223R, K241R, K260R, K278R, or K284R). Cell lysates were prepared under denaturing conditions and subjected to immunoprecipitation with anti-Flag antibody, followed by western blot using anti-HA, anti-Flag, or anti-GAPDH antibodies. **c** 293T cells were co-transfected with Flag-tagged SUMO1 with the indicated wild-type PAX6 and PAX6 variants, containing all lysine-to-arginine mutations, but one or two adjacent lysine (K) sites remained (K28(1), K53(1), K69(1), K89(1), K100(1), K105(1), K148(1), K159(1), K221/223(2), K241(1), K260(1), K278(1), or K284(1)). Cell lysates were prepared under denaturing conditions and subjected to immunoprecipitation with anti-Flag antibody, followed by western blot using anti-HA, anti-Flag, or anti-GAPDH antibodies. Showing K53(1) and K89(1) restored the most apparent expression of SUMO1-modified PAX6. **d** Flag-tagged plasmids encoding wild-type PAX6 (WT) and SUMO1-modified sites that mutated PAX6 (K53R, K89R, and K53/89R) were constructed. 293T cells were co-transfected with Flag-tagged plasmids encoding PAX6 WT, K53R, K89R, and K53/89R, HA-tagged SUMO1 as indicated. Cell lysates were immunoprecipitated by anti-Flag antibody and analyzed by western blot with anti-Flag, anti-HA, or anti-GAPDH antibodies.

Based on the prediction program, GPS-SUMO2.0, no lysine residue of p48 PAX6 protein was definitely scored as the major SUMOylation site. To date, SUMOylation modification has been reported to occur via anchor SUMO on the lysine residues of target protein through covalent bonds^37^. To fully explore the SUMO1 modification sites of PAX6, we listed all the lysine positions (K28, K53, K69, K89, K100, K105, K148, K159, K221, K223, K241, K260, K278, and K284) on the sequence of p48 PAX6 protein (Fig. 5a). To evaluate the contributions of these lysine residues to PAX6 SUMOylation, we first constructed PAX6 mutation plasmids, which separately contained one or two adjacent lysine (K) residues mutant to arginine (R) (K28R, K53R, K69R, K89R, K100/K105R, K148R, K159R, K221/223R, K241R, K260R, K278R, or K284R), and verified their SUMO1 modification. However, the results of western blot revealed that no residue was the main SUMOylated site of PAX6, and SUMO1-PAX6 bonds of K53R, K89R, K100/105R, and K260R variants all seemed to be lower compared with PAX6 WT (line2) (Fig. 5b). Therefore, we further generated another 13 PAX6 constructs, including all lysine (K)-to-arginine (R) mutations, but one or two adjacent lysine residues remained (K28(1), K53(1), K69(1), K89(1), K100(1), K105(1), K148(1), K159(1), K221/223(2), K241(1), K260(1), K278(1), and K284(1)) and then analyzed their SUMOylation. Finally, we observed that the K53(1) (line3) and K89(1) (line5) variants were more apparently present in the SUMO1 modification of PAX6 compared with other PAX6 variants (Fig. 5c). Taken together, K53 and K89 could be the main SUMOylated sites of p48 PAX6 protein.

PAX6 variants with two-site lysine (K)-to-arginine (R) mutations (K53/89R) were also constructed, after which SUMOylation was detected. 293T cells were co-transfected with constructs encoding exogenous SUMO1, together with wild type (WT), K53R, K89R, or K53/89R mutant PAX6. Immunoprecipitation–western blot revealed that the SUMOylation of K53/89R PAX6 (line4) was the most significantly reduced, while SUMOylation of K53R (line5) and K89R (line 6) also showed slightly decreased expression compared with PAX6 WT (line3) (Fig. 5d). To sum up, these results suggested that K53 and K89 residues of p48 PAX6 were the main, but not the entire SUMOylation sites.

### SUMOylation regulated protein stability of PAX6

**Fig. 6:**
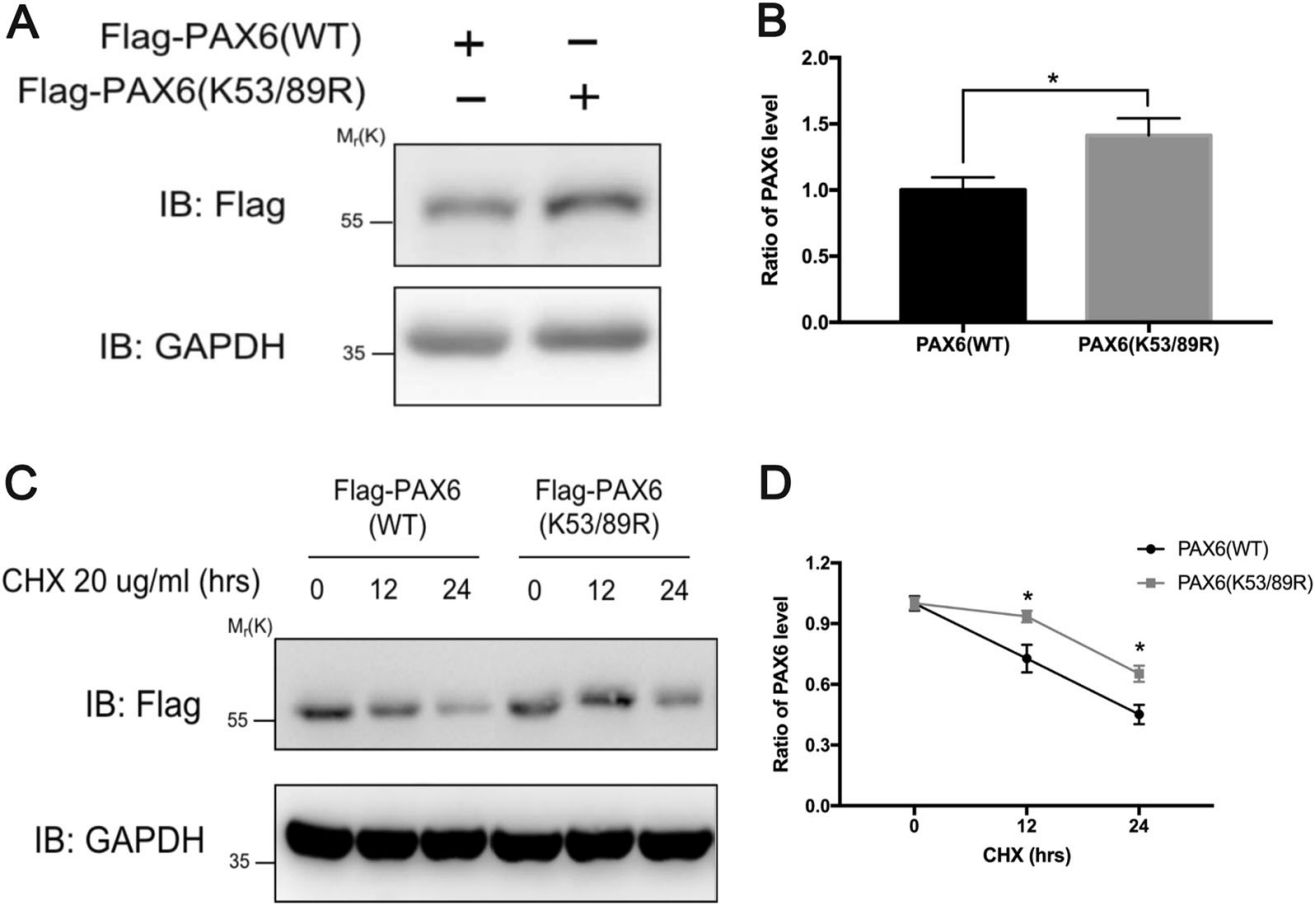
SUMO1 modification changed the expression levels of PAX6 protein in vitro. **a** Analysis of protein expression by western blot. B4G12 cells were transfected with the same amount of Flag-tagged PAX6 WT or PAX6 K53/89R plasmid. Cell lysates were proved with anti-Flag and anti-GAPDH antibodies. **b** Relative expression levels of PAX6 were calculated. Note: de-SUMOylation induced the increased PAX6 protein level. **c** B4G12 cells were transfected with either PAX6 WT or PAX6 K53/89R plasmid, and then treated with 20 μg/ml CHX for 0, 12, or 24 h as indicated inhibiting protein synthesis. Cell lysates were analyzed by western blot using anti-Flag and anti-GAPDH antibodies. **d** The ratio of PAX6 protein was compared. Error bars showed means ± SEM (*n* = 3/group; *p < 0.05).

We separately transfected the same amounts of WT and K53/89R PAX6 plasmids in B4G12 cells. The results of western blot showed that the protein level of SUMOylation-deficient mutant K53/89R PAX6 was significantly higher compared with WT PAX6 (*p* < 0.05), thus indicating that PAX6 SUMOylation modification could regulate the stability of PAX6 protein (Fig. 6a, b). Furthermore, before cycloheximide (CHX) was added to inhibit de novo protein synthesis, a 1.2–1.5-fold amount of WT PAX6 DNA was transfected to achieve a similar expression amount of K53/89R PAX6 protein, which was relatively stable when compared with WT PAX6 protein (*p* < 0.05) (Fig. 6c, d). Taken together, these results demonstrated that de-SUMOylation could stabilize PAX6 protein by decreasing its degradation in B4G12 cells. In other words, SUMOylation modification could reduce the stability of PAX6 protein in vitro.

### Decreased PAX6 protein attributed to aggravated damages in SENP1^+/−^ mice CECs in vivo

**Fig. 7:**
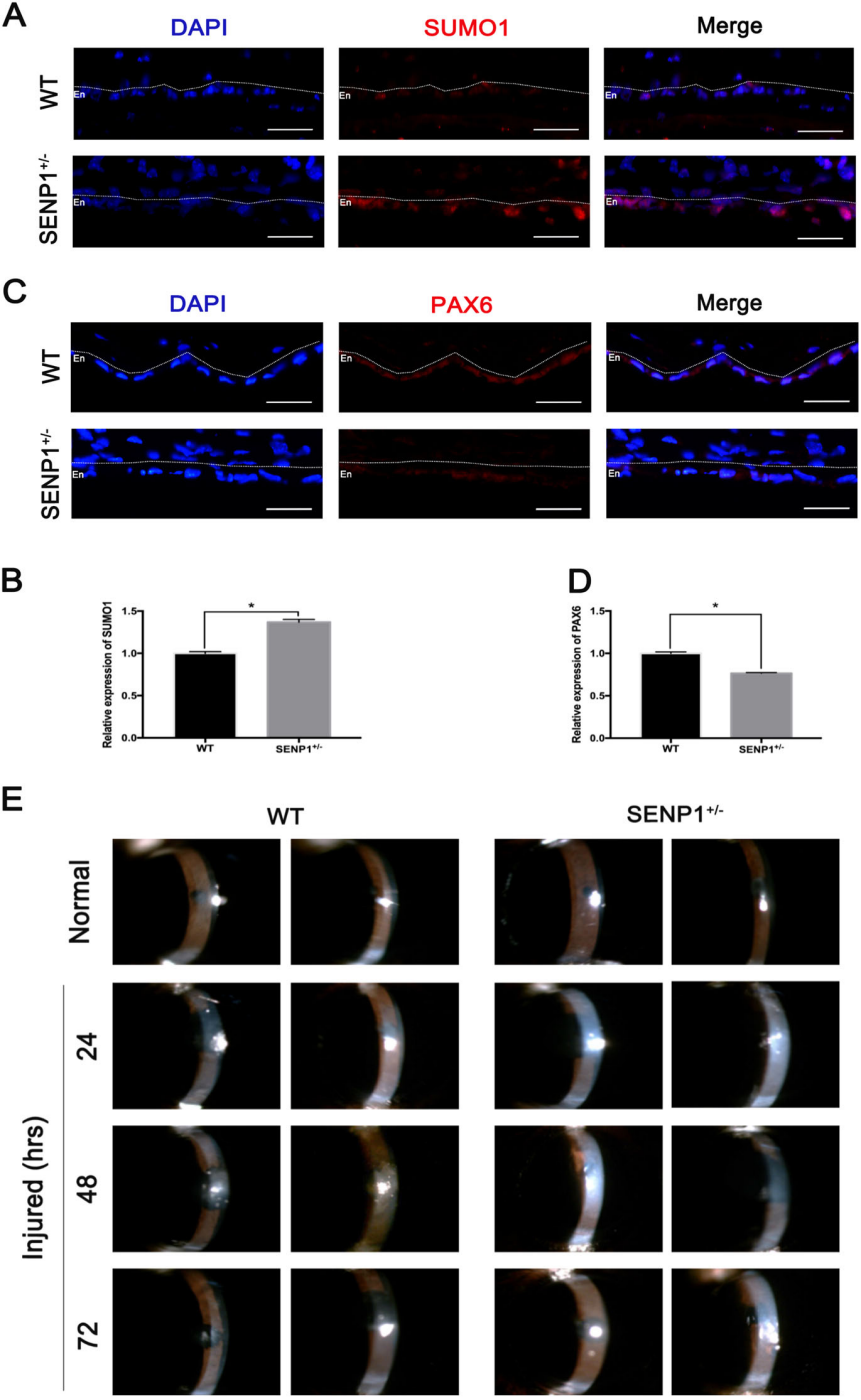

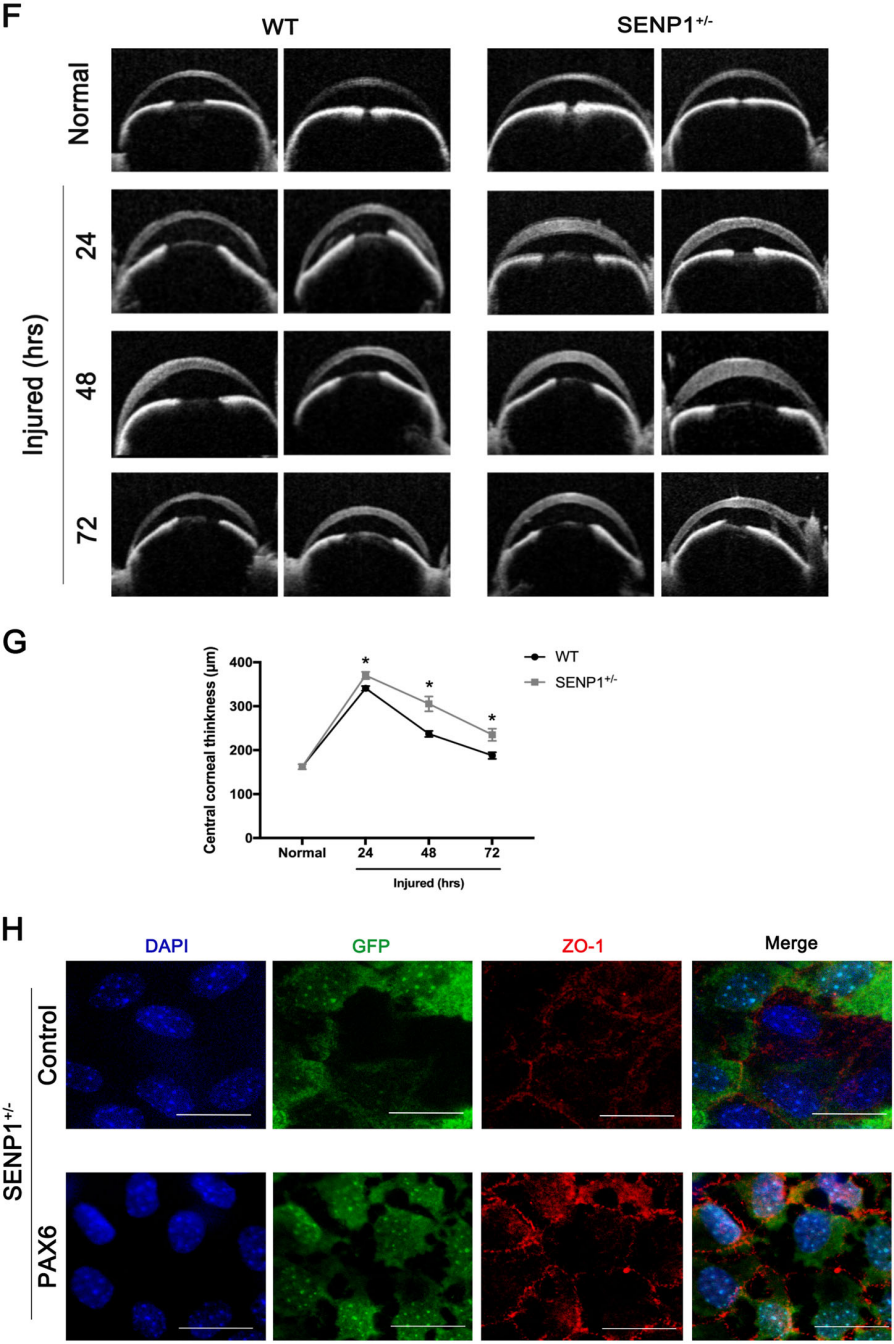
Decreased PAX6 protein expression and aggravated damages in SENP1^+/−^ mice CECs in vivo. **a**, **b** Representative images (**a**) and statistics graph (**b**) presented an expression of SUMO1 in the corneal endothelium of WT and SENP1^+/−^ mice. **c**, **d** PAX6 staining (c) and relative expression of PAX6 (**d**) in WT and SENP1^+/−^ mice CECs was evaluated. The dotted lines highlighted the boundary between the endothelium (En) and the anterior segment. Upregulated SUMOylation induced the downregulation of PAX6 protein in murine corneal endothelium in vivo. **e**, **f** The representative images of a slit lamp (**e**) and OCT (**f**) examinations of SENP1^+/−^ and WT mice showed the normal and injured corneas. **g** Statistics indicated that the averages of CCT of SENP1^+/−^ mice were higher than those of WT mice at 24 h, 48 h, and 72 h after cryoinjury. (**h**) Injured corneas of SENP1^+/−^ mice were acquired for organ culture; representative ZO-1 staining images of corneas of SENP1^+/−^ mice transfected with GFP (Control group) or PAX6 (PAX6 group) were taken. Note: enforced PAX6-protected SENP1^+/−^ mice CECs after injury via improving tight junction among cells. Scale bar = 20 μm. Error bars showed means ± SEM (*n* = 3/group; *p < 0.05).

To verify that the expression levels of PAX6 protein were also regulated by SUMOylation in vivo, we employed corneas of WT and SENP1^+/−^ mice for immunofluorescence staining, as knockout of SENP1 (SENP1^−/−^) is embryonically lethal^37^. As SENP1 was the enzyme of de-SUMOylation, the expression levels of SUMO1 protein in CECs of SENP1^+/−^ mice were significantly higher compared with WT mice (*p* < 0.05) (Fig. 7a, b). Consistent with the previous results, PAX6 protein was slightly present in the normal CECs of WT mice, while there was less PAX6 protein in normal CECs of SENP1^+/−^ mice (*p* < 0.05), as the increased degradation of PAX6 was caused by more SUMOylation (Fig. 7c, d). In addition, as the previous results proved that enforced PAX6 protein had a protective role in wounded rat CECs, we constructed corneal endothelium injury models on WT and SENP1^+/−^ mice to further explore the effects of PAX6 protein and its relation with SUMOylation modification in vivo CECs. The slit-lamp examinations revealed that the corneal opacity of SENP1^+/−^ mice was more serious compared with WT mice at 24, 48, and 72 h after cyroinjury (Fig. 7e). Besides, OCT examinations found more thickened central corneas in SENP1^+/−^ mice compared with corneas of WT mice at 24 h, 48 h, and 72 h after cryoinjury (Fig. 7f, g) (*p* < 0.05). The more opaque and thicker corneas indicated a more severely injured corneal endothelium in SENP1^+/−^ mice compared with WT mice. Furthermore, corneal tissues of SENP1^+/−^ mice after cryoinjury were organ-cultured with the corneal endothelium facing upward for more satisfactory PAX6 transfection efficiency. Then GFP vector (Control group) or GFP-PAX6 (PAX6 group) was transfected, and corneal tissues were collected for ZO-1 staining to detect the injured status of CECs after 24 h of culture. The flat-mounted images showed that CECs transfected with PAX6 exhibited better morphology and expressed more ZO-1, indicating that PAX6 overexpression assisting the corneal endothelium of SENP1^+/−^ mice recovered better in “barrier” function via improving the tight junction (Fig. 7h). Taken together, in CECs of SENP1^+/−^ mice, decreased expression of PAX6 caused by the relatively increased SUMOylation level induced more severe injuries compared with CECs of SENP1 WT mice after injury.

## Discussion

PAX6 is an evolutionally conserved transcription factor in the initial development of the eye^38^. After birth, PAX6 protein nearly disappears in the endothelial layer of the cornea under normal status, which is a reason for current few studies on PAX6 protein in adult CECs^36,39^. Intriguingly, PAX6^+/−^ adult mice manifest more injured corneal endothelium, compared with corneal epithelium^40^. In the present study, we also found relatively normal CECs in the adult corneal rim, which appeared regular and flat, and expressed very little PAX6 protein. In comparison, the expression of PAX6 protein in injured human CECs with irregular morphology and nuclei was significantly increased (Fig. 1). In addition, in vivo PAX6 protein was also apparently upregulated in the universally used model of corneal endothelial cryoinjury in mice and rats (Fig. 2). Enforced expression of PAX6 protein in the corneal endothelium led to less corneal opacity and edema via increasing the “barrier” function of CECs, thus indicating the protective potentials of PAX6 (Fig. 3). Previous studies have also reported that upregulated PAX6 can preserve corneal epithelial identity under pathological conditions, and induce recovery self-renewal of late-passaged limbal niche cells^41,42^. Herein, we identified the protective role of PAX6 in corneal endothelial injuries.

Post-translational modifications of transcription factors, especially SUMOylation, have been addressed by a number of studies^26,43^. Researchers have discovered many modification forms of PAX6, including phosphorylation and ubiquitination by TRIM11^44,45^. Besides, it has been reported that p43 PAX6 is derived from SUMO1-modified p32 PAX6 via the method of in vitro protein synthesis. Also, SUMOylation regulates the transcriptional function of p32 PAX6 in the embryonic lens epithelial cells of human and mouse^18^. In the current study, we focused on the longest isoform of PAX6 (p48 PAX6) to comprehensively identify the possible sites of SUMOylation^46,47^. In our in vitro experiments, we verified that PAX6 could be SUMO1-modified in both endogenous and exogenous systems (Fig. 4), and defined K53 and K89 residues as the main SUMO1-modified sites of p48 PAX6 (Fig. 5). As reported, through the method of ex vivo synthesis, p32 PAX6 is SUMOylated at its K91 residue in the paired-like homeodomain (HD), which is highly conservative among isoforms and referred to the K241 residue of p48 PAX6^18^. The SUMOylation of K241R p48 PAX6 mutant was proved to still exist in Fig. 5b, and K53 and K89 residues, which are not contained in p32 PAX6, were more likely the SUMO sites of p48 PAX6 compared with the K241 residue. SUMOylation attaches to the lysine residue, so that it can participate in the degradation of substrate proteins by competing against or combining with the ubiquitination^48,49^. We found that SUMOylation-deficient PAX6 was more significantly stable in vitro (Fig. 6). More importantly, SENP1^+/−^ mice are naturally fit to provide higher SUMOylated status of PAX6 protein, also stronger degradation of PAX6 protein, for studying the effects of PAX6 SUMOylation on the injured CECs in vivo (Fig. 7a–d)^50^. Our results showed that the lower expression of PAX6 and more severe wounded status of CECs in SENP1^+/−^ mice than WT mice, and exogenous supply of PAX6 in SENP1^+/−^ mice could rescue CECs with better recovery via improving the tight junction (Fig. 7e–h). Similar process has also been reported in Peroxiredoxin-6, another well-studied SUMO substrate protein in the eye. Peroxiredoxin-6 could reverse the injurious process of lens opacity and cell death induced by oxidative stress^51^. Also, mutant Peroxiredoxin-6 at its SUMO1 sites could gain protective functions by maintaining its integrity and activity in epithelial cells of the aging human lens. Recently, SUMOylation has inspired an increasing number of researchers to explore the different target proteins and roles in the development of ocular disease treatments^24,52^. Currently, we focused on p48 PAX6 SUMOylation, including major sites, and SENP1-mediated de-SUMOylation improves the stability of PAX6 and amplifies the protective effects of PAX6 on corneal endothelial injuries. The surgical replacement of the endothelium is the only treatment option for corneal endothelium decompensation with absolute efficiency^6,53^. Considering that demand for donor corneas surpasses the supply, researchers have been trying to uncover the deeper and wider mechanisms of injured CECs so as to provide the base for alternative therapeutic methods to alleviate the injurious process. Adult human CECs cannot proliferate in vivo, and ex vivo culture is passage limitation with EMT risks^2,6^. Moreover, due to their monolayer cellular structural features, adequate corneal endothelial primary cells are not readily available for experiments^1^. Consequently, studies on corneal endothelium are always challenging for researchers. Y-27632, a ROCK inhibitor, has shown the ability to reduce corneal endothelial wounds in animal models by stimulating the proliferation of residual CECs with EMT changes^10,54^. Moreover, Y-27632 treatment still relies on the co-treatment of cultured human CEC transplantation, and is limited to clinical trials due to the difficulty of getting enough CECs^12^. As a result, the development of simple pharmacologic therapies for corneal endothelial diseases is still a matter of utmost urgency^12,54^. According to our findings, we verified that SENP1 induced de-SUMOylation of PAX6 to improve protein stability and have stronger protective effects during the wound process of the corneal endothelium. Therefore, SENP1 protein and SUMOylation-deficient PAX6 have great potentials to become keys of drug development. Future studies are needed to explore more specific protective mechanisms of SUMOylation-deficient PAX6 in injured corneal endothelium and novel therapeutic targets to prevent or treat corneal endothelial diseases.

In summary, our study highlighted the protective roles of PAX6 in the process of corneal endothelium injury, as well as SUMOylation modulation. First, we confirmed the increased expression of PAX6 protein in wounded human monolayer and in murine and rat corneal endothelium injury model. Second, we forced PAX6 expression in corneal endothelium that could alleviate the corneal edema induced by injuries via improving the “barrier” function. In addition, we demonstrated that PAX6 protein could be modified by SUMO1 but not SUMO2/3. Both K53 and K89 residues were the main SUMO1-modified sites of p48 PAX6, and SENP1 acted as a major de-SUMOylating enzyme for p48 PAX6^18,44^. Importantly, we found that de-SUMOylation mediated by SENP1 promoted the expression of PAX6 protein via the amplified stability in vitro and in vivo corneal endothelium. Meanwhile, the protective effects on injured corneal endothelium induced by increased PAX6 protein were confirmed in vivo in SENP1^+/−^ mouse injury models. Therefore, PAX6, as a key factor in corneal endothelial wound healing and SUMOylation-deficient PAX6 protein with higher stability can be potentially applied as therapeutic methods to treat or prevent corneal diseases, and not only endothelial injuries.
